# TRPV1 Channels in the Central Nervous System as Drug Targets

**DOI:** 10.3390/ph17060756

**Published:** 2024-06-07

**Authors:** Loris A. Chahl

**Affiliations:** School of Biomedical Sciences and Pharmacy, University of Newcastle, Callaghan, NSW 2308, Australia; loris.chahl@newcastle.edu.au or loris.chahl@gmail.com; Tel.: +61-408491121

**Keywords:** TRPV1, CNS, capsaicin, neuropsychiatric disorders, schizophrenia, pain

## Abstract

TRPV1 channels are polymodal cation channels located predominantly on primary afferent neurons that are activated by inflammatory mediators, capsaicin (the active component in chili peppers), and noxious heat. TRPV1 channel antagonists are potential new analgesic agents, but their development has been hindered by the finding that they also produce loss of thermal homeostasis and response to noxious heat. Results from recent studies of the TRPV1 channel indicate that it might be possible to develop TRPV1 channel antagonists that inhibit pain without affecting noxious heat sensation. TRPV1 channels are also present in the central nervous system (CNS) and have been implicated in learning, memory, and behaviour. TRPV1 channel modulators have been proposed to have possible therapeutic potential in the treatment of neurological and psychiatric conditions. However, further understanding of the role of TRPV1 channels in the CNS is required before therapeutic advances in the treatment of neuropsychiatric conditions with TRPV1 channel modulators can be made.

## 1. Introduction

The ability to sense noxious and damaging stimuli is essential for the survival of animals. The sensory nerves that transmit nociceptive information to the central nervous system (CNS) are equipped with many receptors and ion channels that respond to a wide variety of stimuli. Of these, the transient receptor potential vanilloid type 1 (TRPV1) channel has attracted intense interest. The TRPV1 channel is a member of the vanilloid subfamily of the mammalian transient receptor potential (TRP) channel superfamily (see [1] for an overview). Of the six members of the vanilloid TRP channels (TRPV1-6), the TRPV1 channel was the first to be cloned and currently has the most extensive pharmacology. The TRPV1 channel is a polymodal, non-selective cation channel, localised on nociceptive primary afferent sensory neurons, which is activated by noxious heat, protons, a variety of inflammatory mediators and chemicals, and most notably by capsaicin, the hot component in chili peppers. Nociceptive primary afferent neurons have both afferent and efferent functions. The activation of TRPV1 channels on primary afferent neurons produces pain (afferent function) and also the release of neuropeptides, including substance P and calcitonin gene-related peptide (CGRP), from the central and peripheral terminals of the neurons (efferent function), which are responsible for antidromic vasodilatation and neurogenic inflammation [2,3]. The cloning of the TRPV1 channel [4] marked a major milestone in pain and sensory nervous system research, which had commenced more than 40 years earlier in Hungary with the studies of the actions of capsaicin by N. Jancsó and Szolcsányi [5]. The subsequent finding that responses to noxious heat and inflammatory pain were impaired in mice lacking capsaicin receptors (formerly known as VR1 receptors) [6] led to the recognition of the TRPV1 channel as a potential new target for the development of analgesic agents. Confirmation that TRPV1 channels are also located in many regions of the CNS (see below) has generated considerable interest in their possible role in neuropsychiatric disorders. This review focuses on the potential of the TRPV1 channel in the CNS as a drug target.

## 2. Capsaicin and TRPV1 Channel Antagonists as Analgesic Agents

An important aspect of the action of capsaicin on TRPV1 channels is that its agonist action is followed by a prolonged period of desensitisation of primary afferent neurons, by mechanisms which are not fully understood [7,8]. This property has led to the use of capsaicin as an analgesic agent despite the initial irritant effect produced by its agonist action [9]. The low efficacy of low-dose capsaicin creams and the irritant side effects of higher-dose formulations have limited the use of capsaicin as an analgesic agent [7]. 

Over the past 20 years since the cloning of the TRPV1 channel, much research attention has focused on the development of TRPV1 channel antagonists as analgesic agents [10]. Several TRPV1 antagonists have been developed, but their clinical usefulness has been curtailed by changes in body temperature regulation, which result in hyperthermia, and by a lack of ability to sense noxious heat, which has led to burn injuries [11]. These effects might have been expected from animal studies, which showed that TRPV1-deficient (*Trpv1*–/–) or capsaicin-desensitised rodents have greatly increased noxious heat threshold responses and exhibit deficiencies in heat-loss mechanisms which cause the development of hyperthermia [6,12,13]. However, other studies, including single-unit electrophysiological studies and thermal pain tests in rats and mice, indicated that TRPV1 channels were not essential for normal noxious heat detection, presumably because other TRP channels such as TRPV2 and TRPA1 play an important role in heat detection in these species [14,15,16,17]. Nevertheless, it now seems that in humans, TRPV1 channels play a major role in noxious heat detection as well as playing an essential role in thermal homeostasis in warm ambient temperatures [11]. As a result of these set-backs, to date, no TRPV1 antagonist has been approved for clinical use, despite intensive drug development research.

Further understanding of the mechanisms involved in the hyperthermic and analgesic responses to TRPV1 antagonists is required to determine whether the development of modality-specific antagonists that produce analgesia but do not produce hyperthermia is a realistic goal. Understanding of the mechanisms by which TRPV1 channels regulate body temperature is incomplete [13]. Nevertheless, the recent study by Yue et al. [18] has clarified that sensory input to the CNS via central projections of nociceptive neurons, rather than direct action in the CNS, is essential for the modulation of core body temperature by drugs that act on TRPV1 channels. Furthermore, Garami et al. [19] demonstrated that the neural pathway of the hyperthermic response involved sensory nerves in trunk muscles. On the other hand, studies with TRPV1 antagonists with differing brain penetration abilities have shown that CNS penetration is necessary for analgesia in pain states induced by central sensitisation [20]. 

## 3. TRPV1 Channels in the CNS

Earlier studies indicated that TRPV1 channels were mainly localised to nociceptive primary afferent neurons, and distribution within the CNS was restricted mainly to the hypothalamus [4,21,22]. However, it is now known that they are widely distributed in the CNS of mice, rats, humans and monkeys [23,24,25,26,27,28,29,30] and in diverse tissues including arteriolar smooth muscle cells [22], kidneys [25] and retinas [31], although at lower levels than in primary afferent neurons [32]. Table 1 shows the distribution of TRPV1 channels in CNS regions of the mouse brain as shown by specific [^3^H]resiniferatoxin binding [28]. High levels of binding were found in the olfactory system, neocortex, hippocampus, thalamic and hypothalamic nuclei, interpeduncular nuclei, periaqueductal grey, locus coeruleus and cerebellum, as well as in the dorsal horn of the spinal cord, dorsal root ganglia and trigeminal ganglia [28]. The discovery of TRPV1 channels in the CNS has led to extensive studies over the past 15 years of their possible roles in learning and memory, behaviour, and animal models of neurological and psychiatric conditions.

TRPV1 channels have been reported to be present in the presynaptic terminals of both excitatory glutamatergic and inhibitory γ-aminobutyric acid (GABA)-ergic neurons in several brain regions where activation enhanced synaptic transmission; see [33]. TRPV1 channels have also been proposed to be located postsynaptically in certain regions including the dentate gyrus and nucleus accumbens [34]. In a peripheral nerve ligation mouse model of neuropathic pain, cortical expression of TRPV1 in neurons was increased, a finding that has led to the suggestion that TRPV1 channels play an important role in central pain states [35]. 

TRPV1 channels are not restricted to neurons but have also been found on glial cells. TRPV1 channels in microglia were found to be localised mainly in mitochondria and have been proposed to enhance glutamatergic transmission in cortical neurons by the production and release of inflammatory mediators [36]. In the mouse anterior cingulate cortex, TRPV1 channels were found to be more highly expressed in microglia than in neurons. TRPV1 channel activation caused microglia to release extracellular microvesicles, thereby enhancing neurotransmission [35]. TRPV1 channels have also been reported to be present in astrocytes, and it has been suggested that this expression is induced by inflammatory mediators in brain injury states and that TRPV1 channels play a role in neuroinflammatory processes in the CNS [36]. 

TRPV1 channels have been found in neurites and growth cones, where they were shown to be involved in the formation of filopodia in neurons [37]. Furthermore, a developmental role for TRPV1 channels has been proposed since TRPV1 channels are expressed in mouse neural precursor cells during postnatal development but not in adult mice [38]. TRPV1-knockout mice have been found to have an increase in neural precursor cells in the dentate gyrus and the subventricular zone, although there was less differentiation into neurons and glia, indicating a disturbance in neuronal growth and differentiation in these animals; see [39]. 

Long-term potentiation (LTP) and long-term depression (LTD) are considered to be involved in memory formation. In brain slices, TRPV1 channel activation by agonists, capsaicin and resiniferatoxin, triggered LTD at excitatory synapses on hippocampal interneurons [40], promoted LTP in hippocampal CA1 neurons and exerted a protective effect against the effects of acute stress on spatial memory [41]. Furthermore, TRPV1-knockout mice had reduced hippocampal LTP compared with wild-type mice, supporting a role for TRPV1 in synaptic plasticity [42]. Subsequent studies in rats have shown that acute administration of a TRPV1 agonist improved cognitive performance in passive avoidance learning tasks [43]. Interestingly, a form of LTP in the striatum and neocortex has been shown to require both TRPV1 channels and cannabinoid CB_1_ receptors [44]. The endogenous cannabinoid, anandamide, can activate TRPV1 channels, and TRPV1 channels have been suggested to act as ionotropic cannabinoid receptors [45]. The complex relationship between TRPV1 channels and the endocannabinoid system remains to be fully explored.

## 4. TRPV1 Channels and Neurological Disorders 

Beneficial effects of capsaicin have been reported in animal models of neurodegenerative diseases and stroke; see [46,47]. In transgenic mice with mutated amyloid precursor protein, a model of Alzheimer’s disease, capsaicin [48] and the selective TRPV1 agonist, vanillin [49], reversed the impairments of hippocampal LTP, spatial learning, and memory. 

In animal models of Parkinson’s disease, capsaicin, acting via TRPV1 channels, reduced neurodegeneration and improved behavioural outcomes, reportedly by reducing oxidants and proinflammatory cytokine production by activated microglia [50]. However, TRPV1 antagonists have also been found to have neuroprotective effects in animal models of Parkinson’s disease (see [47]), indicating that further understanding of the importance of a balance of agonist, antagonist and desensitisation properties of TRPV1 channel modulators is required. 

In animal models of stroke, capsaicin was found to reduce infarct area and improve neurological deficits. The mechanism of this action of capsaicin was proposed to be down-regulation of glutamate NMDA receptors, although other actions such as desensitisation could not be ruled out [51]. In a randomised clinical trial in human stroke patients with dysphagia, capsaicin was found to improve swallowing; see [46]. In contrast, in most studies, capsaicin has been shown to increase seizures in animal models of epilepsy, whereas TRPV1 antagonists reduce seizures; see [46,52]. 

## 5. TRPV1 Channels and Psychiatric Conditions

Anxiety and depression are the most common mental disorders worldwide. The finding that TRPV1-knockout mice exhibited behavioural changes of reduced fear and anxiety compared with wild-type mice [42] has led to increasing interest in the possible role of TRPV1 channels in diverse neuropsychiatric disorders [53,54,55,56]. Studies using several mouse and rat models of anxiety (elevated plus maze, Vogel conflict test, social interaction test, open field) have shown that the TRPV1 antagonist, capsazepine, reduced anxiety; see [56]. Capsazepine, by an action in the periaqueductal grey, also reduced the innate fear response induced in rats exposed to a predator; see [56,57]. In the forced swim test model of depression in rats, TRPV1 antagonists reduced immobility time, indicating an antidepressant effect (see [56]), and interestingly, low-dose capsaicin also produced antidepressant-like effects and enhanced the response to a sub-effective dose of amitriptyline [58]. Thus, the effects of TRPV1 modulation on anxiety, fear and depression are complex and not yet clear.

Addiction is a growing worldwide concern and is the cause of considerable morbidity and psychiatric illness. Therefore, increased understanding of addiction is the focus of considerable pharmacotherapeutic research. TRPV1 channels and CB1 receptors have been found to be colocalised on the cell bodies of neurons in several brain regions implicated in reward and addiction [30]. The role of TRPV1 antagonists in studies of addiction has been reviewed recently [55,56]. In animal models of addiction, TRPV1 antagonists inhibited morphine- and methamphetamine-conditioned place preference. TRPV1 antagonists also inhibited methamphetamine self-administration and inhibited reinstatement of cocaine-seeking behaviour; see [55,56]. 

Schizophrenia is a severe, debilitating mental illness with a lifetime prevalence of about 0.7% in the world [59]. It is characterised by positive symptoms (delusions, hallucinations, thought disorders), negative symptoms (anhedonia, social withdrawal, poverty of thought), and cognitive dysfunction and has strong heritability. Although the overt signs and symptoms of schizophrenia such as psychosis do not usually manifest until early adulthood, recent studies have suggested that cognitive decline precedes the development of psychosis [60]. 

Despite extensive investigation, the aetiology of schizophrenia remains unknown and presents a major challenge to neuroscience and psychiatry. Schizophrenia is considered to result from aberrations in neuronal function, and a large genome-wide association study has implicated multiple genes expressed in CNS neuronal synapses [61]. Epidemiological and genetic studies have suggested that schizophrenia results from disordered neurodevelopment, rather than neurodegeneration, and has its origins in the prenatal or neonatal period [62]. Much research has involved studies of post-mortem human brains, despite the limitations of post-mortem changes and the effects of antipsychotic drug treatments on the subjects prior to death. Two decades ago, several post-mortem studies on human brains reported that the brains of subjects with schizophrenia had reduced volume, larger ventricles, thinner cortices, and increased neuronal density, particularly in the prefrontal and temporal regions, compared with the brains of control normal subjects [63]. These findings led to the ‘reduced neuropil’ hypothesis that the symptoms of schizophrenia result from reduced cortical connectivity rather than a reduction in neuronal numbers [63]. Magnetic resonance imaging (MRI) studies in subjects with schizophrenia have also found reduced cortical grey matter volume in prefrontal temporal and parietal cortex areas, which is consistent with the post-mortem evidence of reduced cortical neuropil [64]. Several studies have shown that the reduction in neuropil is due to reduced dendritic spine density in multiple cortical regions, and factors regulating dendritic development and F-actin stabilisation have been implicated; see [65]. 

Although schizophrenia is considered to be primarily a disorder of neurodevelopment, risk alleles related to apoptotic genes have also been suggested to be associated with schizophrenia. Dendritic pruning and programmed cell death are part of normal brain development. However, aberrations in these mechanisms in schizophrenia have been suggested from studies of post-mortem temporal cortexes from subjects with schizophrenia, where a reduction in Bcl-2 and an increase in the BAX/Bcl-2 ratio, markers indicative of apoptosis, were found; see [66]. Understanding the role of apoptotic mechanisms in schizophrenia is important as it could lead to new treatments for the disorder.

One of the factors limiting the study of schizophrenia is the lack of animal models. Indeed, schizophrenia in all its complexity may be a uniquely human condition. The reports of reduced pain sensitivity and reduced niacin skin flare responses in subjects with schizophrenia prompted a study of the effect of neonatal treatment of rats with capsaicin as a possible animal model of schizophrenia [67]. Systemic treatment of neonatal rats with capsaicin produces a neurotoxic effect in which the animals have a permanent loss of TRPV1 primary afferent neurons [68]. These animals might be expected to exhibit similar behavioural and developmental characteristics to TRPV1-knockout animals. Studies of the effect of neonatal capsaicin treatment on rat brains and behaviour showed that at 5–7 weeks of age, rats treated with capsaicin as neonates had increased locomotor activity in a novel environment compared with vehicle-treated animals [67]. Increased activity in young TRPV1-knockout mice has also been reported [69]. Furthermore, behavioural changes in young rats treated with capsaicin have also been found, with the animals having reduced memory function, as well as disturbances in thermoregulation and motor activity [70]. These observations supported the proposal that some changes induced by neonatal capsaicin treatment mimic those induced by TRPV1 knockout.

A striking finding in rats treated as neonates with capsaicin was that compared with control animals, they had reduced brain weight, reduced coronal and hippocampal cross-sectional areas, reduced cortical thickness and increased neuronal density in several cortical areas [67]. However, the body weight of the capsaicin-treated animals was not significantly less than that of control animals. Some of these changes were found to persist into adulthood [71]. If the loss of TRPV1 channels was responsible for these findings, similar changes might also be expected to be found in TRPV1-knockout animals. Systematic studies of brain weight and regional dimensions do not seem to have been reported in TRPV1-knockout mice. However, in a study of the development of the hippocampal formation in TRPV1-knockout mice, no substantial difference in neuronal migration, morphology, synapse formation or myelination could be detected between the knockout mice and control mice [72]. It should be noted that a major difference between knockout animals and those treated with capsaicin as neonates is that neonatally capsaicin-treated animals have been subjected to a brief but intense stimulation of primary afferent neurons prior to permanent loss of function due to neurotoxicity. This intense stimulation might result in permanent brain changes which would not be seen in knockout animals.

The resemblance between the brain changes found in rats treated with capsaicin as neonates [67] and those found in post-mortem human brains of subjects with schizophrenia [63], together with behavioural changes reported in TRPV1-knockout mice, led Newson et al. [67] to propose that rats treated during early development with capsaicin might be an animal model of schizophrenia. However, this model has not been validated, as sensorimotor gating, measured by prepulse inhibition (PPI), was not affected in these rats [70,71], indicating that the capsaicin-treated rats did not satisfy this most widely accepted criterion for an animal model of schizophrenia. Both the endocannabinoid and vanilloid systems have been implicated in schizophrenia [73], and PPI has been shown to be disrupted in cannabinoid CB_1_ and CB_2_ receptor-knockout mice [74,75]. However, the effect of TRPV1 knockout on sensorimotor gating has not been reported. 

A promising animal model of schizophrenia is the maternal separation model in rats. In this model, the early-life stress of maternal separation leads to behaviours in the adult animals including increased locomotor activity, impairment of PPI inhibition and cognitive deficits, behaviours that have been proposed to be associated with schizophrenia [76]. The adult rats that had been subjected to maternal separation in early life were also found to have significantly increased expression of the pro-apoptotic proteins BAX, caspase3 and cleaved-caspase3 in the prefrontal cortex and hippocampus compared with control animals, and decreased expression of the anti-apoptotic protein Bcl-2, as well as decreased expression of TRPV1 in the prefrontal cortex [77]. A noteworthy finding was that daily treatment of these rats with 1 mg/kg of capsaicin reversed the neuronal and behavioural deficits. These findings led the authors to propose that clinical trials of chili peppers or capsaicin should be undertaken as a treatment for schizophrenia [77]. 

The effects of capsaicin and TRPV1 channel antagonists on neuropsychiatric disorders are difficult to summarise. However, as a broad generalisation, it would seem that brain disorders involving degenerative changes or damage, where there is a deficit in function, might be improved by capsaicin, presumably via an agonist action on TRPV1 channels, whereas disorders where there is a possible increase in the activity of certain brain regions might be improved by TRPV1 antagonists (see Table 2). Studies with capsaicin are fraught with the problem that it is often not certain whether the agonistic action or the desensitisation effect was responsible for the observed responses. Nevertheless, it is clear that any therapeutic use of capsaicin for its agonist action would require careful attention to dosage in order to avoid desensitisation. 

## 6. Therapeutic Potential of TRPV1 Channel Modulators for the Treatment of Pain and Neuropsychiatric Disorders

The challenge of for the development of TRPV1 antagonists is considerable. The goal for the development of TRPV1 antagonists for the treatment of pain is to develop drugs that selectively block the activation of TRPV1 channels by algogenic agents but do not block activation by thermal stimuli. These drugs would ideally have good CNS penetration and act on CNS TRPV1 channels as well as on primary afferent TRPV1 channels, as studies in rats have shown that antagonism of TRPV1 channels in the CNS contributes to broad-spectrum analgesia produced by TRPV1 antagonists [20]. Recent cryo-electron microscopic studies have revealed the mechanisms involved in the polymodal functionality of the TRPV1 channel and have led to an increased understanding of proton modulation of the channel and capsaicin activation [78]. Other recent studies have revealed K710N as a discrete site in the TRPV1 channel gating domain that is crucial for nociception. TRPV1^K710N^-knock-in mice had reduced responses to nociceptive stimuli but had normal responses to heat [79]. Such studies hold promise for the development of TRPV1 channel antagonists as analgesic agents that do not block the response to thermal stimuli.

The challenge for the development of TRPV1 modulators for the treatment of CNS disorders is probably even greater than for the development of analgesic agents. Ideal drugs would act on CNS TRPV1 channels without any, or minimal, effect on primary afferent TRPV1 channels. Although there are known variants of human TRPV1 channels, to date, there seems to be no evidence that the CNS TRPV1 channels differ from primary afferent TRPV1 channels. 

TRPV1 knockout, neonatal capsaicin treatment and TRPV1 antagonists all result in a selective loss of input to the CNS from nociceptive primary afferent neurons. Although undoubtedly perceived to be beneficial in relieving pain and inflammation, the effect of selective loss of nociceptive sensory input to the CNS over a prolonged period of time is unknown. It is well established that a loss of neuronal input produces compensatory neuronal and synaptic plasticity in the CNS. Indeed, TRPV1-knockout mice were found to have increased expression of serotonin 5-HT_1A_ and GABA_Aγ_^2^ receptors and a reduction in the expression of GABA_Aα_^2^ receptors and NR2A-containing NMDA receptors, effects which were proposed to underlie the antidepressant and anxiolytic effects, increased aggression, reduced social interactions and reduced short-term memory observed in these mice [80]. 

The changes in the behaviour of TRPV1-knockout mice [80], and the brain changes found in rats treated as neonates with capsaicin [67], raise the possibility of undesirable behavioural changes and teratogenic effects that might be induced by treatment with TRPV1 channel antagonists. Clearly, the effects of TRPV1 channel antagonists on behaviour and teratogenesis will require extensive investigation before such drugs are approved for human use. 

Although the available evidence from animal studies would suggest that TRPV1 channels are involved in emotional and cognitive behaviours, and a deficit in these channels might result in mental disorders in humans, an important consideration regarding any such therapeutic potential of TRPV1 modulators is the predominant role of TRPV1 channels on primary afferent neurons which might mask other direct effects in the central nervous system. Indeed, the wide distribution of primary afferent neurons with their high density of TRPV1 channels, and the extensive second-order neuronal central projections from their terminals in the spinal cord and medulla, which ultimately reach most levels of the CNS, raise the question as to whether any behavioural and brain changes produced in animals treated with systemic TRPV1 modulators were due to direct effects on central neurons or mediated indirectly by central projections from primary afferent neurons. Although there is now considerable experimental evidence that central TRPV1 channels are active and can produce changes in central neurons in in vitro experiments, the possibility must be considered that these effects might play only a minor role in vivo and might be overshadowed by effects mediated by primary afferent neuronal inputs. The finding of the essential role of primary afferent neurons in thermoregulatory changes induced by TRPV1 antagonists supports such a possibility [19].

Chili peppers have been widely used in many cuisines since ancient times. Therefore, if chili had a marked effect on neuropsychiatric disorders, it might be expected that folklore would have evolved about the possible use of chili peppers for the treatment of such disorders, or conversely, their deleterious effect on such disorders. The fact that such folklore has not arisen, or at least has not been widely propagated, may be attributed to several factors. The different content of capsaicin in the numerous varieties of chili peppers might have precluded any clear indications for the treatment of such complex disorders. Furthermore, the desensitisation produced by capsaicin could potentially confound any clear indication of therapeutic potential due to variable agonist and desensitisation responses. 

A recent cross-sectional study of Chinese college students investigated the relationship between spicy food consumption and psychological health [81]. The results of this study suggested a positive association between frequent spicy food consumption and depressive/anxiety symptoms in adolescents, but no such association was found for stress symptoms. Interestingly, the duration of spicy food intake was not correlated with the odds of having psychological symptoms, which the authors suggested might be due to the desensitisation effect of long-term exposure to capsaicin. In a fifteen-year study of Chinese adults, an association between high chili intake and cognitive decline was found [82]. These findings were in contrast with another study on Chinese adults, where a beneficial effect of a high chili intake was found on blood amyloid-β levels (a biomarker of Alzheimer’s disease) and cognitive function [83]. The current interest in TRPV1 channels will, no doubt, prompt many future controlled studies on the causal relationships between neuropsychiatric disorders and capsaicin consumption.

## 7. Conclusions

Pain is arguably the greatest clinical problem. The ongoing search for new and improved treatments for pain has prompted great interest in the potential of TRPV1 channel antagonists as analgesic agents. The recent intensive investigations of the TRPV1 channel have resulted in considerable advances in the understanding of the functioning of this complex polymodal channel. These studies now hold the promise of the development of new TRPV1 channel antagonist analgesic agents that do not affect the detection of noxious thermal stimuli. However, the effects of TRPV1 channel modulators in the CNS require further investigation, not only to determine the possible usefulness of these agents in CNS disorders but also to determine possible CNS side effects that might be produced by TRPV1 antagonist analgesic agents. 

## Figures and Tables

**Table 1:** Distribution of specific TRPV1 binding in the mouse nervous system. (Adapted from Roberts et al. [28]).

Region	TRPV1 Specific Binding
TELENCEPHALON	
Olfactory system	
- glomerular layer	+++
- olfactory nerve layer	+++
- piriform cortex	+
Neocortex (all regions)	+++
Metacortex	
- retrosplenial cortex	+++
Basal ganglia	
- caudate putamen	+
- nucleus accumbens core	++
- substantia innominata	+
Hippocampal formation	
- CA1 region	++
- CA2 region	+++
- CA3 region	++
- dentate gyrus	++
Amygdala	
- amygdaloid nuclei	++
- lateral septal nucleus	++
Thalamus	
- paracentral thalamic nucleus	+++
- paraventricular thalamic nucleus	+
- nucleus reuniens	++
- ventral posterior thalamic nucleus	+
- ventromedial thalamic nucleus	+
- zona incerta	+
Hypothalamus	
- arcuate hypothalamic nucleus	++
- dorsomedial hypothalamic nucleus	++
- periventricular hypothalamic nucleus	++
- ventromedial hypothalamic nucleus	+
MESENCEPHALON	
- interpeduncular nucleus	+++
- periaqueductal grey	+++
- deep mesencephalic nucleus	+
- raphe nucleus	++
- superior layer of superior colliculus	++
RHOMBENCEPHALON	
- locus coeruleus	++
- olivary complex	+
CEREBELLUM	
- cerebellar cortex	+++
- cerebellar medulla	+
- granular cell layer	++
- molecular cell layer	+
- deep cerebellar nuclei	+
SPINAL CORD	
- dorsal horn	++
TRIGEMINAL GANGLIA	+++
DORSAL ROOT GANGLIA	++

Specific binding was calculated by subtracting the specific [^3^H]resiniferatoxin binding in the nervous system regions of TRPV1-null (*Trpv1−/−*) mice from that of TRPV1 wild-type (*Trpv1+/+*) mice [28].

**Table 2:** Summary of effects of TRPV1 agonists and antagonists on CNS disorders.

TRPV1 Agonist/Antagonist	Disorder	Response
Antagonist—A-784168	Rat model of pain involving central sensitisation	Analgesia in central pain states [20]
Agonist—capsaicin, vanillin	Mouse model of Alzheimer’s disease	Reversed impairment of hippocampal LTP, learning and memory [48,49]
Agonist—capsaicin	Mouse and rat models of Parkinson’s disease	Reduced neurodegeneration and improved behaviour; see [46,50]
Antagonist—AMG9810	Rat model of Parkinson’s disease	Attenuation of motor deficits; see [47]
Antagonist—capsazepine	Mouse model of Parkinson’s disease	Neuroprotective effect; see [47]
Agonist—capsaicin	Rat model of stroke	Reduced infarct volume and improved deficits; see [46,51]
	Human stroke patients	Improved dysphagia; see [46]
Agonist—capsaicin	Rat model of epilepsy	Increased seizures; see [46,52]
Antagonist—capsazepine		Reduced seizures; see [46,52]
Antagonist—capsazepine	Mouse and rat models of anxiety (elevated plus maze, Vogel conflict test, social interaction test, open field)	Reduced anxiety; see [56]
Antagonist—capsazepine	Rat model of fear (exposure to predator)	Reduced fear and stress responses; see [56,57]
Antagonist—capsazepine	Rat and mouse models of depression (e.g., forced swim test)	Antidepressant-like effect; see [56]
Agonist—capsaicin		Antidepressant-like effect and synergism with tricyclic antidepressants [58]
Antagonist—capsazepine, SB366791	Mouse models of addiction (conditioned place reference (CPP), self-administration (SA))	Inhibited morphine CPP; see [55,56]
		Inhibited methamphetamine CPP and SA; see [55,56]
		Inhibited reinstatement of cocaine-seeking behaviour; see [55,56]
Agonist—capsaicin	Rat model of schizophrenia (maternal separation)	Reversal of neuronal and behavioural deficits [77]
